# The Synergistic Effect of Dibenzyldithiocarbamate Based Accelerator on the Vulcanization and Performance of the Silica-Filled Styrene–Butadiene Elastomer

**DOI:** 10.3390/ma15041450

**Published:** 2022-02-15

**Authors:** Magdalena Maciejewska, Anna Sowińska-Baranowska

**Affiliations:** Department of Chemistry, Institute of Polymer and Dye Technology, Lodz University of Technology, Stefanowskiego Street 16, 90-537 Lodz, Poland

**Keywords:** curing characteristics, mechanical performance, styrene-butadiene elastomer, vulcanization, dibenzydithiocarbamate derivative

## Abstract

This work focused on studying the effect of dibenzyldithocarbamate vulcanization accelerator on the curing characteristics and performance of styrene–butadiene elastomer (SBR) filled with nanosized silica. A dibenzyldithocarbamate derivative was applied as an additional accelerator to enhance the efficiency and the rate of sulfur vulcanization in the presence of two other accelerators, i.e., N-cyclohexyl-2-benzothiazole sulfenamide (CBS) and/or 1,3-diphenylguanidine (DPG). Furthermore, the possibility of reducing the amount of zinc oxide (ZnO) and the elimination of CBS and DPG from elastomer compounds using dibenzyldithiocarbamate accelerator was tested. Dibenzyldithocarbamate derivative applied with other accelerators (especially CBS) effectively enhances the efficiency of SBR vulcanization by reducing the optimal vulcanization time and increasing the crosslink density of the vulcanizates despite the lower amount of ZnO. Moreover, vulcanizates with dibenzyldithocarbamate demonstrate higher tensile strength while having a smaller content of CBS or DPG compared to the reference SBR composites. Thus, the synergistic effect of dibenzydithiocarbamate derivative on the vulcanization and performance of SBR was confirmed. Furthermore, dibenzyldithocarbamate derivative enables the amount of ZnO to be reduced by 40% without harmful influence on the crosslink density and performance of the vulcanizates. Finally, it is possible to replace CBS with a dibenzyldithiocarbamate derivative without the crosslink density and tensile strength of the vulcanizates being adversely affected, while improving their resistance to thermo-oxidative aging.

## 1. Introduction

Vulcanization is one of the most frequently used technological processes in the processing of rubber compounds [1]. Over the years, many curing systems dedicated for rubber compounds vulcanization have been developed. The most commonly used curing systems are based on sulfur, peroxides or metal oxides [2,3]. Most of the rubbers widely used in the production of rubber products contain double bonds in macromolecules and therefore are usually vulcanized with sulfur curing systems [4]. A typical sulfur curing system consists of sulfur as a curing agent (crosslinker), accelerators and zinc oxide (ZnO) as a vulcanization activator.

Vulcanization accelerators and activators have a significant influence on the curing parameters, i.e., optimal vulcanization time, vulcanization temperature and scorch time. In addition, accelerators and activators enhance the efficiency of vulcanization by increasing the amount of sulfur, which is consumed to create crosslinks in the elastomer network. Consequently, the crosslink density of the vulcanizates increases, which in turn affects their properties, e.g., tensile properties, modulus, hardness, thermal stability and resistance to thermo-oxidative aging [2,3,5,6]. On the other hand, final properties of the cured rubber depend not only on the crosslink density but also on the structure of crosslinks created during vulcanization.

The structure of crosslinks depends mainly on the content of sulfur and the sulfur/accelerator ratio in the curing system. Concerning the sulfur/accelerator ratio, curing systems are classified as conventional (CV), semi-efficient (semi-EV) and efficient (EV). The CV curing system consists of high sulfur content and low accelerator content and results in longer, mainly polysulfidic crosslinks in the elastomer network. The presence of polysulfidic crosslinks in the elastomer network improves mechanical and dynamic properties but worsens the thermal stability and thermo-oxidative aging resistance of the vulcanizates. Semi-EV curing systems contain an equivalent amount of sulfur and accelerator content. Vulcanizates cured with a semi-EV system are characterized by optimal mechanical properties and thermal stability. On the other hand, the EV curing system has a low sulfur content and high accelerator content. It results in a shorter scorch time, higher cure rate and higher crosslink density compared to CV [7]. An elastomer network with predominantly mono- and disulfidic crosslinks is created during vulcanization with the EV curing system. Consequently, vulcanizates with better thermo-oxidative aging resistance, higher stiffness and poor stress relaxation ability are obtained [5].

The influence of the curing system composition, i.e., the sulfur/accelerator ratio and the type of accelerator used, has been extensively studied for various rubbers [7,8,9,10,11,12]. For example, Formela et al. [10] applied different accelerators to vulcanize reclaimed rubber. The best cure characteristics and mechanical properties were obtained for reclaimed rubber cured with a CV curing system containing sulfenamide accelerators, i.e., N-cyclohexyl-2-benzothiazole sulfenamide (CBS) and N-tert-butyl-2-benzothiazole sulfenamide (TBBS). On the other hand, Marković et al. [9] confirmed the significant influence of accelerator type on the cure characteristics and mechanical properties of rubber blends composed of natural rubber (NR) and chlorosulfonated polyethylene (CSM). The most beneficial influence on the vulcanization and performance of NR/CSM blends had tetramethylthiuram disulfide (TMTD), whereas the lowest activity was determined for 2-mercaptobenzothiazole (MBT). The highest activity of TMTD was also reported by Gobbi et al. [11] for sulfur vulcanization of isobutylene–isoprene rubber compounds. It should be noticed that TMTD is both an accelerator and a sulfur donor, which may increase the content of sulfur in the curing system and consequently the crosslink density of the vulcanizates.

Ghosh et al. [12] confirmed that the sulfur/accelerator ratio and the type of accelerator significantly affected the cure characteristics, vulcanization efficiency and thus mechanical performance of rubber blends composed of raw styrene–butadiene rubber (SBR) and mechanochemically devulcanized SBR. The CV, semi-EV and EV curing systems based on thiurams, thiazoles and sulfenamide accelerators were applied. The best curing characteristics, mechanical performance and aging resistance were demonstrated by rubber composites cured with the sulfenamide-based accelerator, i.e., CBS, especially when semi-EV curing system was used. Moreover, the structure, i.e., the sulfidity, of crosslinks formed during vulcanization, was confirmed to be strongly controlled not only by the sulfur/accelerator ratio but also by the type of accelerator.

The synergistic effect of some accelerators on the cure characteristics and performance of elastomer composites is also well known. Two or more accelerators can be used together to accelerate the vulcanization by providing a synergistic effect on curing. Appropriate selection of these accelerators allows for better control of the vulcanization process and for the obtention of a good balance between safe processing and quick vulcanization of rubber compounds [13,14,15,16,17]. Ahsan et al. [18] reported the synergistic effect of mercaptobenzothiazole disulfide (MBTS), zinc 2-merkaptothiazole (ZMBT) and 1,3-diphenylguanidine (DPG) in sulfur vulcanization of NR compounds. MBTS/DPG combination resulted in the shortest scorch time accompanied with the highest cure rate and crosslink density of the cured composites, whereas NR cured with the MBTS/ZMBT/DPG curing system demonstrated the best mechanical performance. Alam et al. [19] studied the synergistic effect of the binary system of accelerators consisting of thiuram disulfide, i.e., bis(N-benzyl piperazino) thiuram disulfide (BPTD) and MBTS, on the vulcanization of NR compounds. Rubber compounds cured with the BPTD/MBTS curing system exhibited longer optimal vulcanization time compared to NR composites containing TMTD/MBTS. Vulcanizates obtained using the BPTD/MBTS system exhibited mechanical properties similar to those containing TMTD/MBTS, as well as improved heat resistant behavior. Debnath et al. [20] investigated the synergistic effect of different zinc dithiocarbamates in the presence of thiazole-based accelerators in the vulcanization of NR compounds. The highest mutual activity of accelerators was achieved for the combination of zinc dibenzyldithiocarbamate (ZBEC) with MBTS. The synergistic effect was also reported for zinc xanthates and zinc dithiocarbamates [21], which, applied together, allowed for a higher cure rate and significantly better mechanical properties compared to NR composites cured with zinc xanthate.

Therefore, it still seems reasonable to search for the possibility of reducing the amount of ZnO in rubber products by using commercially available auxiliaries, especially those that will not have a detrimental effect on the crosslink density of vulcanizates.

The research works cited above confirmed that many accelerators can exhibit a synergistic effect on vulcanization and thus can act more advantageously when used in combination with each other than as a single accelerator. Dithiocarbamates are one of these accelerators. Therefore, in this work, a multifunctional additive for rubber compounds, namely, Premix Acti8 (Act8) containing dibenzydithiocarbamate derivative, was applied as an additional accelerator in order to boost the vulcanization of the silica-filled SBR compounds. In addition, the possibility of the reduction of the ZnO content in rubber compounds was explored, since ZnO has been classified as very toxic to aquatic life and thus its industrial use must be reduced [22]. Thus, it seems reasonable to search for the possibility of reducing the amount of ZnO in rubber products by using commercially available auxiliaries, especially those that will not have a detrimental effect on the crosslink density of vulcanizates. We also studied the synergistic effect of Act8 in combination with CBS or CBS and DPG accelerators on the cure characteristics and performance of the silica-filled SBR composites. To our knowledge, studies of the synergistic effect of Act8 and sulfenamide accelerators or their combination with DPG have not yet been carried out. This study is a continuation of our previously published work, when Act8 was applied in combination with DPG or a thiazole-based accelerator, i.e., MBT, to vulcanize SBR filled with carbon black. Since the synergistic effect depends on the type of accelerators used [18,23,24], it is justified to examine this effect for Act8 with sulfenamide-based accelerators, which are commonly used to vulcanize SBR compounds in the industry as well as in the research works [25,26,27,28,29]. Moreover, it should be emphasized that the vulcanization of rubber compounds filled with silica is difficult compared to those containing carbon black. This is due to the significant ability of the silica surface to adsorb the curing system. This has a detrimental effect on the crosslinking efficiency and, consequently, on the performance of rubber products. Therefore, it is necessary to look for curing systems that are characterized by high crosslinking efficiency despite adsorption on the silica surface, such as the systems presented in this research work.

## 2. Materials and Methods

### 2.1. Materials

**Figure 1:**
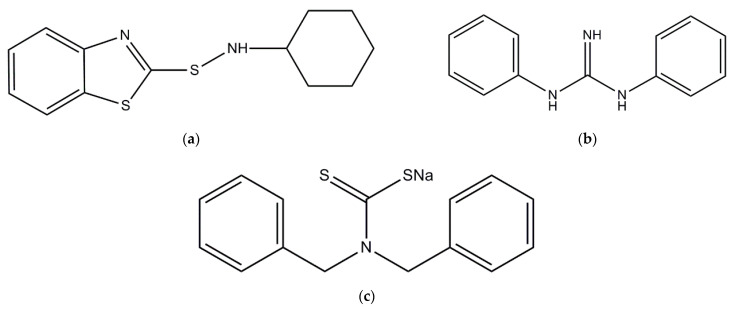
Structures of accelerators used in this study: (**a**) CBS; (**b**) DPG; (**c**) sodium dibenzyldithiocarbamate contained in Act8.

Styrene-butadiene rubber (SBR) of KER1500 type with 23.5% bonded styrene and showing Mooney viscosity ML1 + 4 (100 °C): 50 MU was supplied by Synthos SA (Oswiecim, Poland). It was cured with sulfur (purity 99.9%) provided by Siarkopol (Tarnobrzeg, Poland). 1,3-Diphenylguanidine (DPG, purity 97.0%) and/or N-cyclohexyl-2-benzothiazole sulfenamide (CBS, purity 98.0%) manufactured by Sigma-Aldrich (Poznan, Poland) were applied as primary accelerators. Zinc oxide having a specific surface area of 10 m^2^/g and purity of 99.0% (ZnO; Huta Bedzin, Poland) together with stearic acid (purity 95.0%, Sigma-Aldrich, Poznan, Poland) were applied as standard activators. A dibenzyldithiocarbamate derivative, namely, Premix Acti8, developed by Rubber Nano Products (Pty) Ltd. (Port Elizabeth, South Africa) was used as an additional accelerator. It contains 50% of active substance (sodium dibenzyldithiocarbamate) mixed with poly(ethylene oxide) (PEO) terminated with a silicate compound (sodium metasilicate pentahydrate) to provide its interaction with filler’s surface [30]. Nanosized silica of Aerosil 380 type (Evonik Industries, Essen, Germany) with a specific surface area of 380 m^2^·g^−^^1^, pH 3.7–4.5 and purity of ≥99.8% was applied as a filler. The structures of the accelerators used in this study are shown in Figure 1. In the case of Act8, the structure of the chemical compound, which is active in the vulcanization process, i.e., sodium dibenzyldithiocarbamate, was presented.

### 2.2. Preparation and Characterization of SBR Composites

As the presented work focuses on examining the synergistic effect of accelerators in silica-filled SBR composites, the methodology typically used for elastomer composites was used in the research [31,32,33].

A laboratory two-roll mill (David Bridge & Co, Rochdale, UK), with the roll dimensions: D = 200 mm, L = 450 mm, was employed to prepare SBR compounds. The friction and the width of the gap between the rollers were 1–1.2 mm and 1.5–3 mm, respectively. The rotational speed of the front roll during compounding was 16 min^−^^1^. SBR compounds were prepared following a two-step procedure. First, a masterbatch was manufactured containing the rubber, sulfur and the filler silica Aerosil 380. Then, the masterbatch was divided into eight equal pieces, and the rest of ingredients were introduced into each of these pieces following the recipes shown in Table 1 as parts per hundred of rubber (phr). The number of primary and secondary accelerators referred to the similar ratio used in the industry for SBR composites. Moreover, the sulfur/accelerator ratio has been selected to obtain curing systems of different efficiencies, i.e., CV, semi-EV and EV.

The SBR rubber compounds were cured at 160 °C up to a 90% increase in torque. The optimal vulcanization time (t_90_) was determined using the MDR 2000 Moving Die Rheometer produced by Alpha Technologies (Heilbronn, Germany). The rheometric measurements were carried out following the procedure described in the standard ISO 6502 [34].

The range of SBR curing temperatures and the enthalpy of this process were explored employing a differential scanning calorimeter DSC1 (Mettler Toledo, Greifensee, Switzerland). A small piece of rubber compound with a mass of approximately 10 mg was hermetically sealed in aluminum crucible with a capacity of 40 µL and placed in the DSC1 analyzer cell. The sample prepared was cooled down to –100 °C before the measurement and stabilized isothermally at this temperature for 5 min. After starting the measurement, the sample was heated from –100 to 250 °C in an argon atmosphere (gas flow 20 mL/min) at a heating rate of 10 K/min. The onset curing temperature was determined using STARe software (Version 10, 2010, Mettler Toledo, Greifensee, Switzerland) according to the procedure specified in the ISO 11357-1 [35] standard as the onset temperature of the exothermic peak corresponding to the curing reactions.

An equilibrium swelling method was adopted to investigate the crosslink density of the SBR vulcanizates according to the ISO 1817 [36] standard procedure using toluene as a solvent. Four pieces weighing in the range of 20–30 mg were used for swelling for each of the vulcanizates. The samples were swollen for 48 h at ambient temperature. The crosslink density was calculated based on the Flory–Rehner equation [37], adopting the Huggins parameter of the elastomer-solvent interaction (*χ*) given by Equation (1) [38], where *V_r_* is the elastomer’s volume fraction in the swollen gel.

*χ* = 0.37 + 0.56 *V_r_*. (1)

The tensile properties of the SBR vulcanizates were explored according to the procedure described in the ISO 37 [39] standard. A universal testing machine Zwick Roell 1435 (Zwick Roell, Ulm, Germany) was employed for measurements. Five dumb-bell specimens with a test length of 20 mm and a width of 4 mm were investigated from each of the vulcanizates, and then the mean value of the determinations was taken as the result.

The Shore A hardness was measured for three disc-shaped specimens from each of the SBR vulcanizates. Measurements were carried out according to the ISO 868 [40] standard procedure using a microcomputer-controlled Zwick Roell 3105 (Zwick Roell, Ulm, Germany) hardness tester.

The dynamic mechanical properties of the SBR vulcanizates were studied as a function of temperature using a DMA/SDTA861e (Mettler Toledo, Greifensee, Switzerland) analyzer. Measurements of the dynamic moduli, i.e., storage modulus (E′), loss modulus (E″) and their ratio tan δ, were accomplished in a tension mode over the temperature range of –100 to 80 °C (heating rate of 3 K/min). Specimens of the vulcanizates in the shape of cuboids with a length 10.5 mm, width 4 mm and thickness of approximately 1 mm were oscillating stretched with a frequency of 1 Hz and a strain amplitude of 10 µm. The glass transition temperature (T_g_) of the SBR was determined as the temperature of the maximum of tan δ = f(T) plot, where tan δ is the mechanical loss factor and T is the measurement temperature.

The thermo-oxidative aging of the SBR vulcanizates was carried out following the procedure described in ISO 188 standard [41]. Plates of the vulcanizates with a thickness of approximately 1 mm were stored in a drying chamber (Binder, Tullingen, Germany) at a temperature of 100 °C for 7 days. Next, their tensile properties, i.e., tensile strength and elongation at break, as well as their crosslink density and hardness, were studied and compared to the data determined for the non-aged samples. Finally, the aging factor (A_f_) was calculated according to the procedure described in [42].

Thermogravimetric analysis was employed to explore the thermal stability of the SBR vulcanizates. A TGA/DSC1 (Mettler Toledo, Greifensee, Switzerland) analyzer was adopted to perform measurements for small pieces of the vulcanizates with a mass of approximately 12 mg placed in an opened alumina crucible with a capacity of 70 µL. First, the sample was heated in the temperature range of 25–600 °C in an argon atmosphere (gas flow 40 mL/min, heating rate 20 K/min). Next, the measurement atmosphere was changed into air and the sample was heated to 700 °C (gas flow 40 mL/min, heating rate 20 K/min) to burn the residues after thermal decomposition.

## 3. Results and Discussion

### 3.1. Influence of the Dibenzyldithiocarbamate Accelerator on the Curing Characteristics and the Crosslink Density of the SBR Composites

Several modifications of the composition of the examined SBR rubber compounds were carried out, such as: addition of a variable amount of the dibenzyldithiocarbamate accelerator, reduction of the amount of ZnO, reduction of the amount and elimination of CBS. Each of these modifications concerned the composition of the curing system, and it was therefore crucial to study their influence on the curing characteristics of the SBR composites.

The rheometric properties of SBR compounds were investigated to determine the influence of curing system modifications on the curing parameters. The rheometric measurements were performed at 160 °C. The results are presented in Table 2.

The minimum torque (S_min_) during rheometric measurement corresponds to the viscosity of the uncured rubber compound [43]. The S_min_ values of the tested SBR compounds were comparable taking into account the measurement error. As expected, the composition of the curing system had no significant effect on the S_min_ and hence the viscosity of the uncured rubber compounds.

On the other hand, the torque increment (ΔS) during rheometric measurement results from the increase in the stiffness of the elastomer composite due to vulcanization. Therefore, it refers to the degree of the elastomer’s crosslinking [43]. For SBR compounds containing 2.5 phr of CBS, the ΔS increased with the content of Premix Acti8 (Act8), despite the level of ZnO being reduced by 40% compared to the rubber compound with 5 phr of ZnO. Furthermore, Act8 allowed the content of CBS to be reduced by 40% without detrimental effect on the ΔS. This was due to the beneficial influence of dibenzyldithiocarbamate, which contributed to the increase in the crosslinking degree of the elastomer. It was confirmed by the values of the vulcanizates crosslink density (ν_t_) determined by their equilibrium swelling in toluene.

Regardless of the amount of ZnO and CBS used, vulcanizates with Act8 exhibited significantly higher ν_t_ compared to those without Act8. Furthermore, increasing the content of Act8 from 2 phr to 4 phr had no considerable influence on the crosslink density of SBR vulcanizates, since the values of ν_t_ fluctuated within the measurement error.

As expected, the dibenzyldithiocarbamate derivative beneficially affected the optimal vulcanization time (t_90_) of SBR compounds. The benchmark 2.5CBS/5ZnO demonstrated t_90_ of approximately 26 min. In case of the benchmark, the curing system can be classified as semi-efficient (semi-EV) since the content of sulfur and accelerator in the rubber compound is similar. Applying Act8 reduced the t_90_ of SBR compounds to approximately 7 min. Since Act8 contain 50% of the active substance, i.e., dibenzyldithiocarbamate derivative, the total content of accelerators in SBR compounds with Act8 became higher than the sulfur content, and the curing system became efficient (EV). This resulted in a higher cure rate and a consequently shorter t_90_ as compared to semi-EV.

Despite the accelerating influence on t_90_, applying 2–3 phr of Act8 did not significantly affect the scorch time of SBR compounds (t_02_). On the other hand, when 4 phr of Act8 was used, t_02_ decreased from 3.5 min to approximately 1 min. However, it should be noticed that the content of CBS in a rubber compound with 4 phr of Act8 was 1 phr lower (CBS content of 1.5 phr) than in the benchmark (CBS content of 2.5 phr). Therefore, the decrease in t_02_ was due to both the increased content of Act8 and the reduced by 40% content of CBS. Sulfenamides, like CBS, provide a delayed action to the vulcanization process. They retard vulcanization by increasing the t_02__,_ and, consequently, they ensure the safe processing of rubber compounds [44]. A positive influence of CBS on t_02_ was confirmed by Formela et al. for reclaimed ground tire rubber [10]. Thus, the complete removal of CBS from the rubber compounds had a detrimental effect on the ΔS of the SBR compounds and the crosslink density of the vulcanizates. The removal of CBS from rubber compounds extended the t_90_ compared to SBR cured in the presence of CBS. After CBS removal, the content of sulfur and accelerator (dibenzylditiocarbamate derivative) in 3ZnO/4Act8 vulcanizate became similar, so the curing system applied became semi-EV again.

Most importantly, it was concluded that, despite using the dibenzyldithiocarbamate accelerator, the presence of CBS was crucial to obtaining a good balance between the safe processing (sufficiently long t_02_) and fast vulcanization (short t_90_) of the SBR filled with nanosized silica Aerosil 380. It was more advantageous to use the CBS/Act8 combination than each of these accelerators separately. Thus, the synergistic effect of CBS and Act8 in the vulcanization was confirmed.

It should be noticed that the benchmark containing CBS and DPG exhibited significantly higher ΔS and consequently higher ν_t_ than the reference SBR compound without DPG (2.5CBS/5ZnO). Therefore, the beneficial effect of DPG on the efficiency of the silica-filled rubber compounds vulcanization was confirmed. A similar effect of DPG was postulated by other researchers. As mentioned, according to Jin et al., DPG facilitated vulcanization of the silica-filled rubber compounds due to the preferential adsorption on the silica surface, which reduced the adsorption of the primary accelerator and increased the efficiency of the vulcanization [45]. The strong ability of DPG to be adsorbed on the silica surface was confirmed by Zaborski and Donet [46]. Moreover, it is well known that sulfur vulcanization prefers alkaline conditions. Due to basic nature, DPG may increase the alkalinity of the vulcanization environment and thus the efficiency of the vulcanization [47].

Regarding SBR compounds with CBS and DPG, the influence of Act8 on the curing characteristics was quite positive and not so pronounced as for rubber compounds containing CBS without DPG. Act8 had no significant impact on the S_min_ and, consequently, on the viscosity of uncured SBR compounds compared to the benchmark CBS/DPG/5ZnO. Regardless of the content of ZnO and Act8, SBR composites exhibited quite similar ΔS and, consequently, crosslink density comparable to the benchmark without Act8 (the data varied within the range of experimental error).

Owing to the presence of the dibenzyldithiocarbamate accelerator, Act8 shortened both the t_02_ and t_90_ compared to the rubber compound without Act8. Moreover, the higher the Act8 content, the shorter the t_02_ and t_90_. However, the influence of Act8 on the t_90_ was not as significant as in SBR compounds containing CBS without DPG. This was probably due to the performance of DPG. According to Bosch [30], Act8 was designed to interact with the silica surface in order to reduce the adsorption of the curing system. DPG being preferentially adsorbed on the silica surface [48,49] blocks the active centers on the surface of this filler, thus decreasing its ability to interact with Act8. Hence, the beneficial effect of Act8 on the curing characteristics and vulcanization efficiency is less evident as compared to rubber compounds containing CBS alone.

On the other hand, Act8 consists of poly(ethylene oxide) (PEO) terminated with a silicate compound to provide for its interaction with a filler surface [30]. Thus, in the case of SBR composites without DPG, Act8 can interact with the silica and can be adsorbed on its surface, significantly reducing the adsorption of the curing system. This improves the curing characteristics and crosslink density of the SBR composites.

It should be noticed that Act8 allowed the level of ZnO to be reduced by 40% without detrimental effect on the t_90_ of rubber compounds and the ν_t_ of the vulcanizates. Most importantly, it was proven that, due to the synergistic effect, the use of Act8 seemed to be more advantageous in a curing system with another accelerator, i.e., CBS or CBS/DPG, than as a primary accelerator.

**Figure 2:**
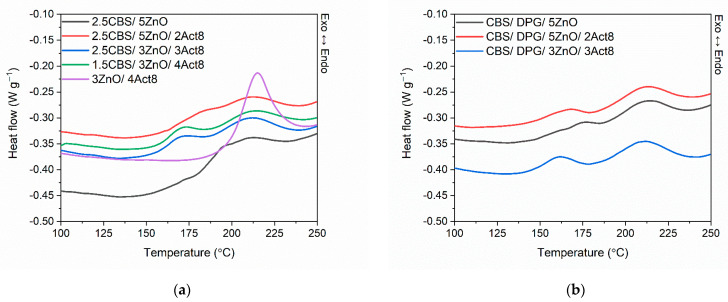
Differential scanning calorimetry (DSC) curves of SBR compounds containing: (**a**) CBS; (**b**) CBS and DPG.

Having established the impact of the dibenzydithiocarbamate derivative on the curing characteristics of SBR compounds, a differential scanning calorimetry (DSC) was employed to study the influence of the curing system composition on the enthalpy and temperature of crosslinking reactions. The DSC curves for SBR compounds are presented in Figure 2, and the results are summarized in Table 3.

Analyzing the DSC curves presented in Figure 1, the crosslinking of SBR compounds was observed as an exothermic process in the temperature range dependent on the composition of the curing system.

Regarding SBR compounds with CBS, crosslinking of the benchmark without Act8 proceeded in the temperature range of 178–232 °C, with the enthalpy of crosslinking (∆H_cross_) being approximately 8.4 J/g. The peak corresponding to crosslinking was rather broad, and the crosslinking proceeded as a one-step process, although the peak was very wide and it was difficult to clearly define a peak temperature. Applying Act8 reduced the onset temperature of crosslinking (T_onset_) by 15–18 °C and slightly increased the ∆H_cross_ compared to the benchmark without Act8 (2.5CBS/5ZnO). Thus, the crosslinking started at a lower temperature and proceeded with a higher energetic effect as compared with the benchmark. On the other hand, the analysis of the DSC curves revealed that, in the presence of Act8, the crosslinking became a two-step process, with one step immediately following the other. The crosslinking peak temperature has shifted to approximately 209 °C, so the second step of crosslinking became dominant.

It should be noticed that the removal of CBS significantly increased the T_onset_ and the enthalpy of crosslinking compared to SBR compounds with CBS. Moreover, crosslinking of the SBR compound without CBS (3ZnO/4Act8) proceeded as a one-step process in the temperature range of 199–230 °C, with a T_peak_ of approximately 214 °C, similarly to the second crosslinking step of the SBR compounds containing CBS. The crosslinking peak was definitely narrower and more intense than the peaks obtained for SBR cured with CBS. Therefore, it was concluded that the first step of crosslinking that occurred in the DSC curves of SBR containing CBS was mainly due to the induction of crosslinking caused by the delayed action of CBS. Then, the second and predominant step of crosslinking resulted from the action of CBS boosted by the addition of fast accelerator, i.e., dibenzyldithiocarbamate derivative. The removal of CBS and the use of dibenzyldithiocarbamate as the primary accelerator eliminated the slow induction of crosslinking, which was characteristic for CBS. Consequently, the crosslinking was induced quickly at high temperatures and proceeded intensively immediately after induction.

At the same time, the higher the temperature of crosslinking, the higher the probability of some post-curing reactions [50], which may deteriorate the efficiency of the vulcanization and, consequently, the final crosslink density of the elastomer network. Thus, the crosslink density of the vulcanizate without CBS, i.e., 3ZnO/4Act8, was significantly lower compared to CBS-containing vulcanizates.

Most importantly, DSC studies revealed that, considering the crosslinking temperature, Act8 together with CBS demonstrated a synergistic effect on the vulcanization of SBR composites filled with nanosized silica.

As expected after the analysis of the rheometric properties, applying Act8 did not significantly affect the range of the crosslinking temperatures of rubber compounds cured with CBS and DPG. Regardless of the composition of the curing system, crosslinking proceeded as a two-step process in the temperature range of 145–234 °C, with the predominant second step occurring at a temperature above 180 °C (T_peak_ of approximately 211 °C). The enthalpy of crosslinking increased slightly with the content of Act8 in rubber compounds. It resulted from the slight increase in the intensity of the second crosslinking step. The results of the DSC analysis confirmed that applying Act8 had a more pronounced influence on the crosslinking of SBR compounds with CBS as the primary accelerator compared to those containing CBS and DPG. This is in a good agreement with the results of rheometric measurements.

The synergistic activity of CBS and dithiocarbamate accelerators was studied by Alam et al. [24] in relation to the zinc dithiocarbates (ZDC) of various structures. Act8 contains sodium dibenzyldithiocarbamate (Figure 1), but its synergistic activity with CBS may be presumed to have similar reasons as the synergistic activity of CBS and zinc dithiocarbamates. Using high-performance liquid chromatography (HPLC), Alam et al. [24] proved that CBS/ZDC systems generated MBT and TMTD during vulcanization accompanied by the formation of cyclohexyl amine from CBS. At the same time, MBT exhibited strong synergistic activity with TMTD, which was confirmed by previous researchers [51,52]. The possible path for the mutual activity of CBS and dithiocarbamate accelerator was presented in [24], whereas possible routes for the synergistic activity of CBS with TMTD, which can be generated from ZDC, were described in [53].

### 3.2. Influence of the Dibenzyldithiocarbamate Accelerator on the Tensile Properties and Hardness of the SBR Composites

**Figure 3:**
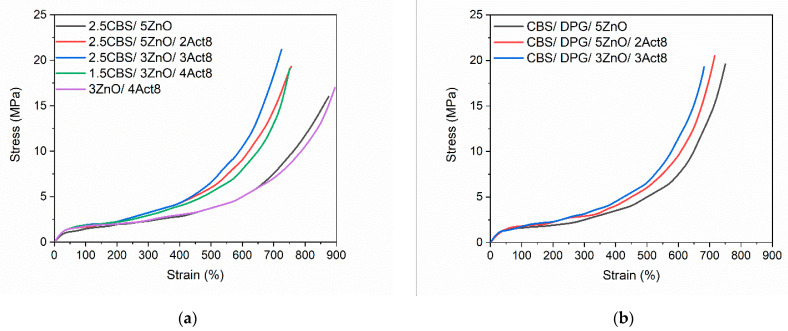
Stress-strain curves of SBR vulcanizates containing: (**a**) CBS; (**b**) CBS and DPG.

Mechanical properties of the cured rubber, such as tensile strength, modulus at relative elongation and hardness, strongly depend on the crosslink density [54,55]. Thus, having established that the curing system composition affected the crosslink density of the vulcanizates, we than studied their tensile properties and hardness. The results are given in Table 4, whereas the stress–strain curves are shown in Figure 3.

The Young’s modulus (E) presented in Table 4 is a measure of the stiffness of the material and depends on the crosslink density of elastomer [56]. As the stiffness of the tested vulcanizates increased with the crosslink density, their Young’s modulus was also increased. Hence, vulcanizates containing Act8 in combination with CBS or CBS and DPG were characterized by higher Young’s modulus compared to the samples without Act8. Thus, it can be concluded that the elastic strain resulting from the application of a given stress for Act8-containing vulcanizates was smaller than for elastomers without this accelerator [57].

Regarding the stress at 300% relative elongation (Se_300_), the effect of the curing systems on Se_300_ fully correlated with their influence on the crosslink density of the vulcanizates. Owing to the higher crosslink density, vulcanizate containing CBS and Act8 exhibited higher Se_300_ compared to the benchmark without Act8. Furthermore, due to the similar crosslink density, the vulcanizate with eliminated CBS (nO/4Act8) demonstrated Se_300_ similar to the benchmark 2.5CBS/5ZnO. As far as SBR cured with CBS and DPG is concerned, applying Act8 and reducing the ZnO level did not significantly alter the Se_300,_ similarly to the crosslink density of the vulcanizates.

As expected, the change in the crosslink density of the vulcanizates due to the curing system composition strongly affected their tensile strength (TS). SBR vulcanizate cured with CBS reached the TS of 16 MPa, whereas vulcanizate with CBS and DPG showed significantly higher TS (22 MPa) due to considerably higher crosslink density. In the case of CBS-containing vulcanizates, applying Act8 increased TS by approximately 3–5 MPa. The highest TS of approximately 21 MPa was exhibited by the vulcanizate containing 3 phr of Act8. As mentioned, the complete removal of CBS from rubber compounds significantly decreased the crosslink density of the vulcanizate. Consequently, the TS of 3ZnO/4Act8 vulcanizate was approximately 2–3 MPa lower compared to that of vulcanizate containing CBS. Thus, Act8 and CBS were confirmed to have a synergistic effect on the mechanical properties of SBR vulcanizates. Moreover, Act8 allowed the content of ZnO and CBS in SBR composites to be reduced without detrimental influence on their TS.

Regarding the vulcanizates with CBS and DPG, the curing system composition had no significant influence on the TS. Regardless of the content of Act8 and ZnO, the TS of the vulcanizates ranged from 19.3 MPa to 20.5 MPa, and the differences were within the range of measurement error.

The elongation at break (E_b_) of the vulcanizates is strongly dependent on the crosslink density. The higher the content of crosslinks in the elastomer network, the lower the mobility of the elastomer chains and, consequently, the lower the E_b_ is. In the case of the tested vulcanizates, the influence of the curing systems on E_b_ fully correlated with their effect on the crosslink density. Thus, due to the lowest ν_t_, the highest E_b_ was demonstrated by the benchmark 2.5CBS/5ZnO and the vulcanizate without CBS, i.e., 3ZnO/4Act8 (875% and 895%, respectively). On the other hand, applying Act8 in CBS-containing vulcanizates reduced the E_b_ by approximately 120–150% compared to the benchmark without Act8. It resulted from the highest ν_t_ of the Act8-containing vulcanizates. Owing to the higher ν_t_, the reference vulcanizate with CBS and DPG exhibited an E_b_ of 750%, approximately 125% lower compared to the benchmark without DPG, i.e., 2.5CBS/5ZnO. Act8 reduced the E_b_ of SBR cured with CBS and DPG to 682%. Nevertheless, it can be considered that the SBR vulcanizates containing Act8 showed very good flexibility.

Considering the standard deviation of the results, the curing system composition did not significantly alter the hardness of the SBR vulcanizates. The benchmark with CBS exhibited the hardness of 61 Shore A, whereas Act8-containing vulcanizates demonstrated slightly higher hardness of approximately 62–63 Shore A. The lowest hardness of 60 Shore A was exhibited by the vulcanizate without CBS, i.e., 3ZnO/4Act8, which showed that the lowest ν_t._ Hardness of the SBR cured in the presence of CBS and DPG was in the range of 62–64 Shore A, quite similar to that of CBS-containing vulcanizates. Most importantly, despite the reduced content of CBS and ZnO, SBR vulcanizates were characterized by similar hardness.

### 3.3. Influence of the Dibenzyldithiocarbamate Accelerator on the Dynamic Mechanical Properties of the SBR Composites

**Figure 4:**
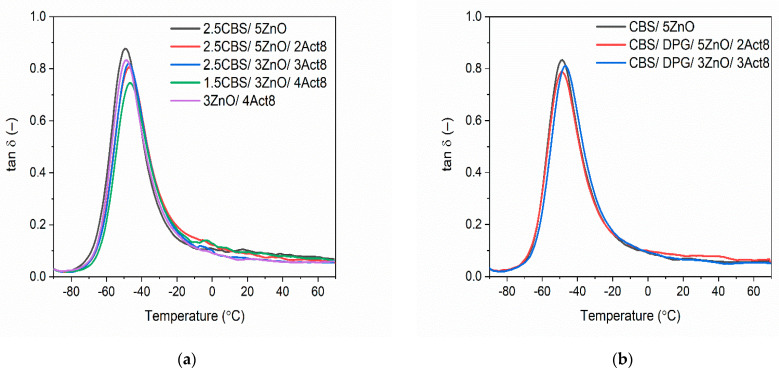
Mechanical loss factor (tan *δ*) curves versus temperature of SBR vulcanizates containing: (**a**) CBS; (**b**) CBS and DPG.

Dynamic mechanical analysis (DMA) was adopted to conduct the measurements of the viscoelastic properties of SBR vulcanizates as a function of temperature. The impact of the curing systems on the glass transition of the SBR elastomer and its dynamic mechanical behavior in the glassy state and in the rubbery elastic region was explored. The curves of the mechanical loss factor tan δ as a function of temperature were determined. The results are presented in Figure 4 and summarized in Table 5.

The DMA curves of SBR vulcanizates showed in Figure 4 revealed the glass transition of SBR elastomer, which occurred in the temperature range of −70 °C to −15 °C, as confirmed by the peak of the mechanical loss factor (tan δ) on the DMA curves. The temperature of the maximum of tan δ peak corresponds to the glass transition temperature (T_g_) of SBR elastomer. Thus, the T_g_ of SBR determined for the benchmarks without Act8 was approximately −51 °C and −49 °C for the vulcanizate with CBS and CBS/DPG, respectively. The T_g_ of SBR determined for Act8-containing vulcanizates ranged from −48 °C to −47 °C, which was slightly higher compared to that of the benchmarks. It resulted from the higher crosslink density of SBR cured in the presence of Act8. T_g_ strongly depends on the mobility of polymer chains, which is affected by the presence and the number of crosslinks. The higher the number of crosslinks, the smaller the mobility of the polymer chains and, consequently, the higher the T_g_. On the other hand, vulcanized rubbers are characterized by a low crosslink density compared to highly crosslinked polymers such as epoxy resins and polyurethanes. In weakly crosslinked polymers, a relatively small increase in the crosslink density, as was observed for tested vulcanizates, does not significantly increase the T_g_, in contrast to highly crosslinked polymer matrices [58]. Thus, the T_g_ of SBR determined for the vulcanizates containing CBS and Act8 was only 3–4 °C higher compared to the benchmark 2.5CBS/5ZnO. Curing systems containing CBS and DPG did not significantly alter the T_g_ of SBR, which ranged from −49 °C for the benchmark without Act8 to −47 °C for the vulcanizate containing 3 phr of Act8. Most importantly, taking into account the range of changes in T_g_, Act8 used together with CBS and DPG should not affect the operating temperature of the SBR composites.

The ratio of the loss and the storage moduli (E″/E′) is known as the mechanical loss factor tan δ. It is a measure of the energy dissipated as heat by the polymer during each deformation cycle. Thus, it corresponds to the damping properties of the material [59]. The highest values of tan δ at T_g_ were exhibited by the benchmark 2.5CBS/5ZnO without Act8 and by the vulcanizate with removed CBS, i.e., 3ZnO/4Act8 (tan δ of approximately 0.94 and 0.88, respectively). It resulted from the lowest crosslink density and, consequently, the highest mobility of the elastomer chains in these vulcanizates as compared to those containing CBS and Act8.

Regarding SBR vulcanizates containing CBS as the primary accelerator, the addition of Act8 significantly reduced the height of the tan δ peak. This was due to the decreased elastomer chains mobility resulting from the higher crosslink density of Act8-containing vulcanizates. Thereby, the values of tan δ at T_g_ were in the range of 0.74–0.82. The same relationship between the crosslink density of the vulcanizates and the values of tan δ was confirmed by Ahankari et al. [60] and Sombatsompop et al. [61] for SBR composites and NR composites, respectively. Since the crosslink densities of SBR vulcanizates containing both CBS and DPG were similar to those of SBR cured in the presence of CBS and Act8, they demonstrated quite similar tan δ at T_g_, which was in the range of 0.76–0.83.

Analyzing the values of tan δ in the rubbery elastic region at temperatures of 25 °C and 60 °C, respectively (Table 5), it was observed that the composition of the curing system did not significantly alter the damping properties of SBR composites (considering the measurement error). Regardless of the content of Act8 and ZnO or the primary accelerator used (CBS or CBS/DPG), the values of tan δ in the rubbery elastic region were in the range of 0.06–0.09 and 0.05–0.08 at 25 °C and 60 °C, respectively. Moreover, the damping properties of studied SBR composites were stable in the rubbery elastic region, since the values of tan δ did not change significantly as a function of temperature above 10 °C.

### 3.4. Influence of the Dibenzyldithiocarbamate Accelerator on the Thermo-Oxidative Aging Resistance of the SBR Composites

**Figure 5:**
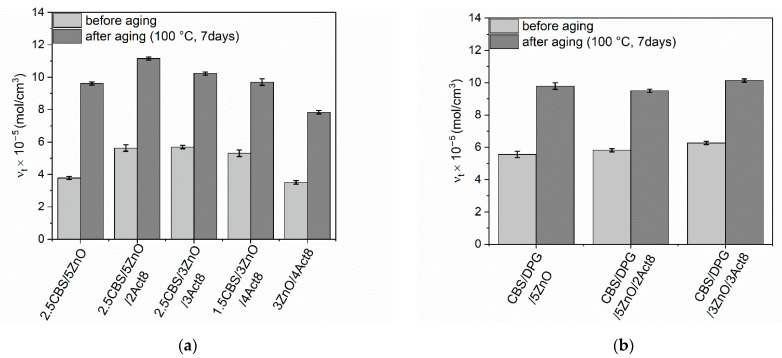
Influence of thermo-oxidative aging on the crosslink density of SBR vulcanizates containing: (**a**) CBS; (**b**) CBS and DPG.

**Figure 6:**
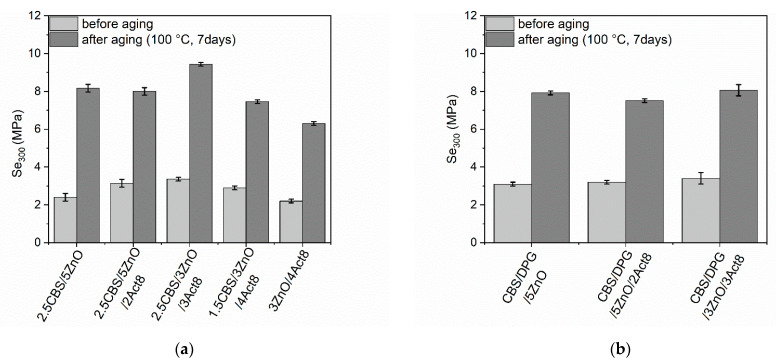
Influence of thermo-oxidative aging on the stress at 300% relative elongation (Se_300_) of SBR vulcanizates containing: (**a**) CBS; (**b**) CBS and DPG.

**Figure 7:**
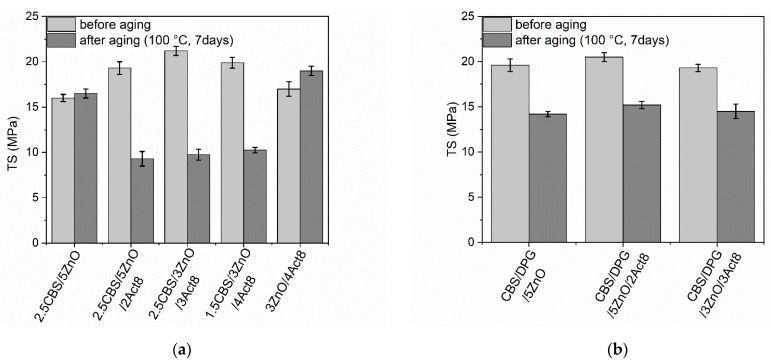
Influence of thermo-oxidative aging on the tensile strength (TS) of SBR vulcanizates containing: (**a**) CBS; (**b**) CBS and DPG.

**Figure 8:**
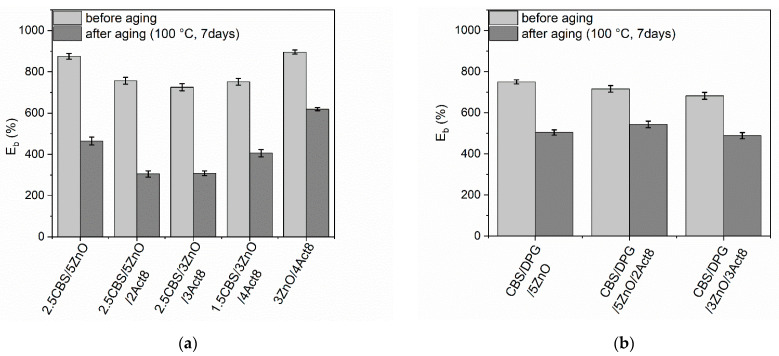
Influence of thermo-oxidative aging on the elongation at break (E_b_) of SBR vulcanizates containing: (**a**) CBS; (**b**) CBS and DPG.

**Figure 9:**
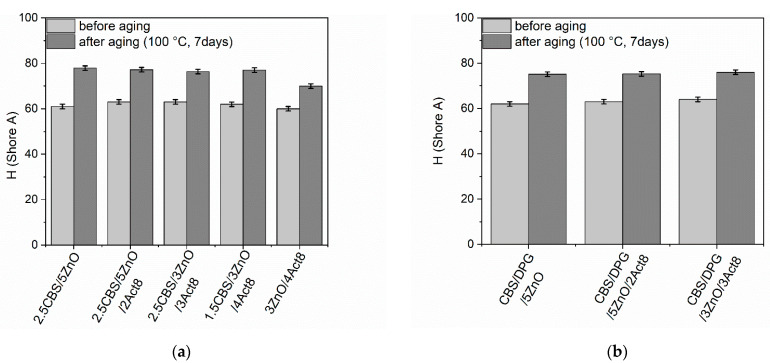
Influence of thermo-oxidative aging on the hardness of SBR vulcanizates containing: (**a**) CBS; (**b**) CBS and DPG.

Rubber products are often used in outdoor applications or at elevated temperatures. Therefore, it was justified to investigate the influence of the curing system composition on the resistance of SBR composites to thermo-oxidative aging. The influence of prolonged thermo-oxidation on the tensile properties, hardness and crosslink density of the SBR vulcanizates was explored, and the results are presented in Figure 5, Figure 6, Figure 7, Figure 8 and Figure 9.

As shown in Figure 5, prolonged exposure to elevated temperature during thermo-oxidative aging induced further crosslinking of SBR composites. It resulted in the considerable enhancement of their ν_t_. A similar influence of prolonged thermo-oxidation on the ν_t_ of elastomers was observed by Nabil et al. [62], Kruželák et al. [63] and Rezig et al. [64].

The highest increase in ν_t_ values was observed for SBR cured with CBS, especially for the benchmark without Act8, i.e., 2.5CBS/5ZnO. This could be due to the delayed action of DPG in the elastomer crosslinking. DPG provides a slow crosslinking rate during the vulcanization of rubber compounds. The same action of DPG is likely to be observed during crosslinking reactions induced by the prolonged thermo-oxidation. Moreover, DPG is an aromatic diamine. This group of chemical compounds, e.g., N-phenyl-1,4-phenylenediamine (NPPD), demonstrates antioxidant activity, which could have contributed to a better resistance of DPG-containing vulcanizates to thermo-oxidative aging [65].

An increase in the ν_t_ of the vulcanizates due to thermo-oxidative aging was the main cause of observed changes in the hardness and mechanical properties, i.e., Se_300_, TS and E_b_. Regardless of the curing system used, SBR vulcanizates exhibited significantly higher Se_300_ compared to non-aged samples (Figure 6). Moreover, during prolonged exposure to high temperatures, the polysulfide linkages in the elastomer network broke down and the monosulfide linkages were formed, which were responsible for higher Se_300_ modulus [66].

Owing to the significantly increased ν_t_, the TS of most vulcanizates after prolonged thermo-oxidation was considerably lower compared to that of non-aged material (Figure 7). The greatest deterioration of TS was observed for the vulcanizates containing CBS and Act8. The TS of aged vulcanizates was reduced by approximately 10–11 MPa compared to that of non-aged samples. On the other hand, thermo-oxidative aging had no significant impact on the TS of the benchmark without Act8, i.e., 2.5CBS/5ZnO. It should be noticed that this vulcanizate demonstrated significantly lower ν_t_ before aging than Act8-containing samples. The TS of cured rubber is commonly known to increase with ν_t_ to a certain critical value of the ν_t_, above which the vulcanizate becomes over-crosslinked. When ν_t_ becomes too high, the average molar mass of the elastomer chain between the two adjacent crosslinks decreases. This restricts the mobility of the chain segment, thus reducing the number of effective network chains. Consequently, the ability of the elastomeric network to transfer stresses is reduced. The over-crosslinked vulcanizate becomes brittle and its TS is reduced [62,67]. Since the non-aged benchmark 2.5CBS/5ZnO exhibited significantly lower ν_t_ compared to vulcanizates with Act8, an increase in ν_t_ of this vulcanizate due to the aging process did not deteriorate the TS. The same effect of thermo-oxidative aging on TS was observed for the 3ZnO/4Act8 vulcanizate, which was characterized by ν_t_ similar to the benchmark 2.5CBS/5ZnO. In contrast, CBS and Act8-containing vulcanizates exhibited high ν_t_ before aging, so a further increase in ν_t_ due to prolonged thermo-oxidation caused them to be over-crosslinked. Consequently, vulcanizates with CBS and Act8 showed significantly lower TS after the aging process as compared to the TS of non-aged samples.

Regarding SBR cured with CBS and DPG as accelerators, thermo-oxidative aging reduced the TS of the vulcanizates by approximately 5 MPa regardless of the Act8 application. Thus, the TS reduction due to aging was 50% lower compared to the vulcanizates without DPG. This resulted from the lower increase in the ν_t_ of the vulcanizates containing CBS and DPG as compared to SBR cured with CBS. Therefore, the beneficial effect of DPG on the resistance of SBR to thermo-oxidative aging was confirmed. The better resistance on the thermo-oxidative aging of elastomers cured with DPG as compared to other accelerators was reported by Travas-Sejdic et al. [68] for NR vulcanizates. Moreover, Ahsan et al. [18] reported that DPG used to activate other accelerators imparted the high aging resistance of rubbers.

The increase in the ν_t_ due to prolonged thermo-oxidation significantly affected the E_b_ of SBR vulcanizates, especially those cured with CBS without DPG. The E_b_ of these vulcanizates was reduced by 350–450% compared to that of non-aged material (Figure 8). This resulted from the restricted elastomer chains mobility by the significantly increased number of crosslinks in the aged elastomer network. As expected, DPG-containing vulcanizates showed a considerably lower reduction in E_b_ compared to SBR cured with CBS without DPG. The E_b_ of these vulcanizates was decreased by approximately 170–250% compared to non-aged samples. This confirmed the better resistance on the prolonged thermo-oxidation of DPG-containing composites.

Regardless of the curing system composition, SBR vulcanizates exhibited significantly higher hardness after thermo-oxidative aging as compared to non-aged samples (Figure 9). Owing to the crosslink density increase, the hardness of the vulcanizates increased by 10–17 Shore A. A slightly higher increase in hardness was observed for the vulcanizates containing CBS without DPG, especially for the benchmark 2.5CBS/5ZnO. The same effect of thermo-oxidative aging on the hardness of SBR vulcanizates was reported by Rezig et al. [64], Ghosh et al. [69] and Mostafa et al. [70].

Having explored the influence of prolonged thermo-oxidation on the tensile properties of SBR vulcanizates, the thermo-oxidative aging factor (A_f_) was determined based on the changes in the TS and E_b_ resulting from the aging process. The results are listed in Table 6. A_f_ can be considered as a quantitative measure of the material’s resistance to thermo-oxidative aging. Since A_f_ is calculated as the ratio of the (TS × E_b_) of the vulcanizates after aging to the corresponding parameters of the non-aged samples [42], the higher the A_f_, the better the resistance of the material to thermo-oxidative aging. The A_f_ value close to 1 is the most preferred because it means that the thermo-oxidative aging does not significantly alter the mechanical properties of the elastomer composite.

The analysis of the A_f_ values collected in Table 6 showed that SBR vulcanizates were susceptible to thermo-oxidative aging. However, their resistance to aging was dependent on the curing system used, particularly the presence of CBS, the amount of Act8 and the use of DPG.

Regarding SBR cured with CBS as the primary accelerator, the highest A_f_ of approximately 0.55 was determined for the benchmark without Act8, i.e., 2.5CBS/5ZnO. Vulcanizates containing Act8 exhibited significantly lower A_f_ compared to the benchmark, which was in the range of 0.19–0.28. As mentioned, owing to the significant increase in the crosslink density, Act8-containing vulcanizates after thermo-oxidative aging became over-crosslinked, and, consequently, their TS and E_b_ were significantly reduced compared to those of non-aged specimens. Thus, the synergistic effect of Act8 on the resistance of SBR to prolonged thermo-oxidation was not observed.

Act8 did not significantly affect the A_f_ of SBR cured with CBS and DPG. The A_f_ of the vulcanizates containing CBS and DPG was in the range of 0.49–0.56. Interestingly, the highest A_f_ of approximately 0.77 was shown by the vulcanizate 3ZnO/4Act8. Similarly, to the benchmark 2.5CBS/5ZnO, the non-aged 3ZnO/4Act8 vulcanizate was characterized by a much lower crosslink density than the other samples. Hence, an increase in the crosslink density due to thermo-oxidative aging did not cause this vulcanizate to be over-crosslinked but improved its TS compared to non-aged material.

### 3.5. Influence of the Dibenzyldithiocarbamate Accelerator on the Thermal Stability of the SBR Composites

**Figure 10:**
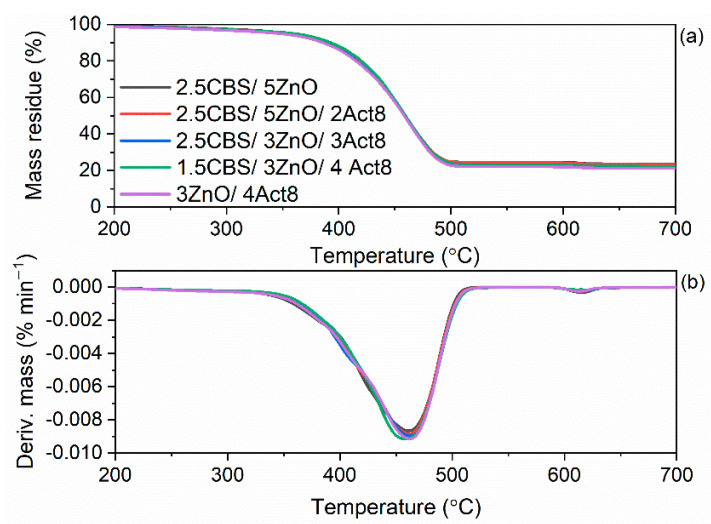
Thermogravimetric (TG) and Derivative Thermogravimetric (DTG) curves of SBR vulcanizates containing CBS: (**a**) TG curves; (**b**) DTG curves.

**Figure 11:**
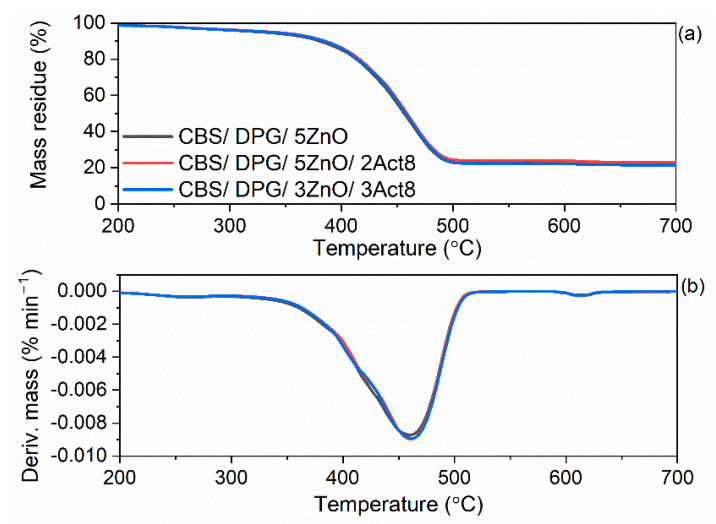
TG and DTG curves of SBR vulcanizates containing CBS and DPG: (**a**) TG curves; (**b**) DTG curves.

Act8 consists of organic components such as PEO and sodium dibenzyldithiocarbamate. Similarly, CBS and DPG accelerators are organic compounds. All of these additives can decompose when heated to high temperatures, deteriorating the thermal stability of SBR composites. Therefore, the impact of the curing system composition on the thermal stability of SBR vulcanizates was explored by thermogravimetry (TG). The results are presented in Figure 10 and Figure 11 and summarized in Table 7. The temperature at which the mass of studied samples was reduced by 5% in relation to their initial mass (T_5%_) was determined as the onset temperature of thermal decomposition.

Following the collection of the data presented in Table 7, it was concluded that DPG had the most significant impact on the thermal stability of SBR composites. Vulcanizates containing DPG as one of the accelerators exhibited approximately 20 °C lower T_5%_ temperature compared to those without DPG and were thermally stable up to a temperature of approximately 330 °C. On the other hand, SBR cured with CBS without DPG demonstrated T_5%_ in the range of 350–358 °C.

**Figure 12:**
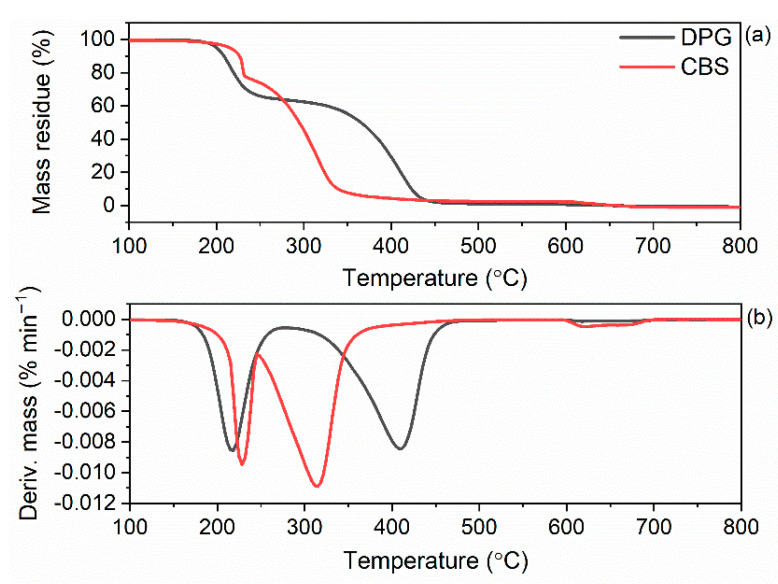
TG and DTG curves of pure CBS and DPG: (**a**) TG curves; (**b**) DTG curves.

The lower T_5%_ of DPG-containing vulcanizates resulted from the lower thermal stability of DPG compared to CBS. It was confirmed by the results of the TG analysis of pure DPG and CBS accelerators (Figure 12, Table 8). The T_5%_ determined for DPG was 198 °C, whereas the T_5%_ of CBS was 219 °C. Regarding the TG analysis of DPG, the results obtained are in a good agreement with those reported by Hu et al. [71]. The thermal decomposition of DPG was reported to begin at a temperature above 160 °C and proceed in two steps. The first step, with a DTG peak temperature (T_DTG_) of approximately 220 °C, corresponded to escaping of aniline, whereas the second step, with a T_DTG_ of approximately 420 °C, was due to the pyrolysis of the stable intermediates from the first decomposition step, e.g., the products of the partial oligomerization of aniline. Due to the first decomposition step, DPG lost approximately 36% of its mass.

On the other hand, the thermal decomposition of CBS started at 195 °C. The T_DTG_ of the first decomposition step of CBS was approximately 240 °C, and the corresponding mass loss was approximately 27%. According to Samide et al. [72], this resulted from the loss of adsorbed water overlapped with the loss of cyclohexylamine due to its evaporation and/or decomposition. The second decomposition step of CBS occurred in the temperature range of 250–350 °C, with a T_DTG_ of approximately 329 °C, and resulted mainly from the decomposition of the benzothiazole part of CBS. Moreover, Samide et al. [72] reported that CBS could be contaminated with 2,2′-dibenzothiazole disulfide (an impurity from the manufacturing process), which may decompose at this step as well.

Regardless of the primary accelerators used, Act8 did not significantly alter the T_5%_ and thus the thermal stability of SBR vulcanizates. The reduction of ZnO content and the removal of CBS had no considerable influence on SBR thermal stability as well. Moreover, the composition of the curing system did not significantly alter the T_DTG_, i.e., the temperature at which the thermal decomposition of the material proceeded at the highest rate.

Analyzing the TG curves of the tested vulcanizates, the main mass loss occurred in the temperature range of 25–600 °C in argon atmosphere and ranged from 75.3% to 77.9%. It resulted from the pyrolysis of the elastomer and the organic components of the rubber compounds, i.e., vulcanization accelerators and the organic part of Act8.

As expected, the lowest mass loss in the temperature range of 25–600 °C was determined for the benchmark 2.5CBS/5ZnO due to the lowest content of organic additives. On the other hand, the highest ∆m_25–600 °C_ was observed for SBR cured with CBS, DPG and 3 phr of Act8 owing to the highest content of organic compounds. The mass loss in the temperature range of 600–700 °C (∆m_600–700 °C_) occurred in an air atmosphere and was of approximately 1%. Since the examined vulcanizates were filled with silica, ∆m_600–700 °C_ corresponded to the combustion of the residue after pyrolysis of the elastomer matrix and organic additives. The residue at 700 °C was in the range of 21.2–23.6% and resulted from the presence of silica used as a filler, zinc oxide, which was used as a vulcanization activator, and ash [73].

## 4. Conclusions

In this work, the influence of the curing system composition, i.e., the type and the amount of the primary accelerator (CBS and DPG), the use and the content of the dibenzyldithiocarbamate accelerator (Act8) and the content of ZnO on the curing characteristics and performance of SBR composites filled with nanosized silica was explored. Most importantly, the composition of the curing system was proved to have a significant influence on the vulcanization parameters and properties of the SBR composites.

Dibenzyldithocarbamate derivative (Act8) applied with other accelerators, i.e., CBS and DPG, effectively increased the efficiency of SBR vulcanization by reducing the optimal time and temperature of vulcanization and by increasing the crosslink density of the vulcanizates despite the lower level of ZnO. The most beneficial influence of Act8 was achieved when CBS without DPG was used as a primary accelerator. Furthermore, applying Act8 in CBS-containing vulcanizates significantly enhanced their tensile strength. Thus, the synergistic effect of Act8 with CBS on the vulcanization and mechanical performance of SBR composites filled with nanosized silica was proven. In addition, Act8 enabled the level of ZnO to be reduced by 40% without detrimental impact on the curing characteristics, crosslink density and performance of the SBR composites. Furthermore, it was possible to replace CBS with an Act8 without deterioration of the crosslink density and tensile strength of the vulcanizates. Moreover, in the case of the 3ZnO/4Act8 vulcanizate, a significant improvement in the resistance to thermo-oxidative aging was observed despite the significantly lower level of ZnO. Thus, Act8 can be successfully used to enhance the properties of elastomer composites containing nanosized silica as a filler.

Additionally, the beneficial effect of DPG on the crosslink density, mechanical properties and resistance to thermo-oxidative aging of the SBR vulcanizates was confirmed.

## Figures and Tables

**Table 1:** Recipes of the SBR rubber compounds, parts per hundred of rubber (phr).

Ingredient (phr)	Reference Samples		Rubber Compounds with Act8					
	REF 1	REF 2	SBR1	SBR2	SBR3	SBR4	SBR5	SBR6
SBR	100	100	100	100	100	100	100	100
Sulfur	2	2	2	2	2	2	2	2
CBS	2.5	1.5	2.5	2.5	1.5	₋	1.5	1.5
DPG	-	2	-	-	-	-	2	2
ZnO	5	5	5	3	3	3	5	3
St. A.	1	1	₋	₋	₋	₋	-	-
A380	30	30	30	30	30	30	30	30
Act 8	₋	-	2	3	4	4	2	3

SBR, styrene–butadiene elastomer; CBS, N-cyclohexyl-2-benzothiazole sulfenamide; DPG, 1,3-diphenylguanidine; ZnO, zinc oxide; St.A., stearic acid; A380, silica Aerosil A380; Act8, Premix Acti8.

**Table 2:** Curing characteristics of the SBR compounds at 160 °C and the crosslink density of the vulcanizates determined using the equilibrium swelling in toluene (S_min_, minimum torque; S_max_, maximum torque; ∆S, torque increase; t_02_, scorch time; t_90_, optimal vulcanization time; ν_t,_ crosslink density).

Composites	S_min_ (dNm)	ΔS (dNm)	t_02_ (min)	t_90_ (min)	$\nu t_{\;}\times 10{-}^{5}\;$ (mol/${\mathrm{cm}}^{3}$)
Rubber compounds with CBS					
2.5CBS/5ZnO	6.6 ± 0.6	19.4 ± 0.6	3.5 ± 0.2	25.6 ± 1.1	3.8 ± 0.4
2.5CBS/5ZnO/2Act8	7.0 ± 0.5	20.8 ± 0.5	3.4 ± 0.2	10.4 ± 0.9	5.6 ± 0.6
2.5CBS/3ZnO/3Act8	6.7 ± 0.5	22.5 ± 0.5	3.0 ± 0.2	7.8 ± 0.8	5.7 ± 0.4
1.5CBS/3ZnO/4Act8	6.9 ± 0.4	21.7 ± 0.4	1.0 ± 0.1	7.1 ± 0.8	5.3 ± 0.3
3ZnO/4Act8	7.3± 0.6	17.1 ± 0.6	0.8 ± 0.1	38.5 ± 1.1	3.5 ± 0.4
Rubber compounds with CBS and DPG					
CBS/DPG/5ZnO	6.9 ± 0.4	22.0 ± 0.4	4.7 ± 0.3	8.2 ± 0.9	5.6 ± 0.4
CBS/DPG/5ZnO/2Act8	6.2 ± 0.6	24.5 ± 0.6	2.9 ± 0.2	6.9 ± 0.6	5.8 ± 0.6
CBS/DPG/3ZnO/3Act8	7.0 ± 0.5	23.0 ± 0.5	1.9 ± 0.2	5.2 ± 0.6	6.3 ± 0.5

**Table 3:** Crosslinking temperature and enthalpy (ΔH_cross_) of SBR compounds determined by differential scanning calorimetry (DSC).

Composites	Temperature of Crosslinking			∆H_cross_ (J/g)
	T_onset_ (°C)	T_endset_ (°C)	T_peak_ (°C)	
	Rubber compounds with CBS			
2.5CBS/5ZnO	178 ± 1	232 ± 1	196 ± 1	8.4 ± 1.2
2.5CBS/5ZnO/2Act8	163 ± 2	236 ± 2	209 ± 2	9.8 ± 1.0
2.5CBS/3ZnO/3Act8	160 ± 2	235 ± 2	209 ± 2	11.0 ± 1.0
1.5CBS/3ZnO/4Act8	158 ± 1	237 ± 1	210 ± 1	10.2 ± 1.1
3ZnO/4Act8	199± 2	230 ± 2	214 ± 2	14.7 ± 0.9
	Rubber compounds with CBS and DPG			
CBS/DPG/5ZnO	147 ± 1	233 ± 1	211 ± 1	8.0 ± 1.2
CBS/DPG/5ZnO/2Act8	147 ± 1	234 ± 1	211 ± 1	8.6 ± 1.0
CBS/DPG/3ZnO/3Act8	145 ± 2	234 ± 2	210 ± 2	9.8 ± 0.9

**Table 4:** Tensile properties and hardness of SBR vulcanizates (E, Young’s modulus; Se_300_, stress at 300% relative elongation; TS, tensile strength; E_b_, elongation at break; H, hardness).

Composites	E (MPa)	Se_300_ (MPa)	TS (MPa)	E_b_ (%)	H (Shore A)
Vulcanizates with CBS					
2.5CBS/5ZnO	1.9 ± 0.1	2.4 ± 0.1	16.0 ± 0.4	875 ± 14	61 ± 1
2.5CBS/5ZnO/2Act8	2.2 ± 0.1	3.1 ± 0.1	19.3 ± 0.1	756 ± 8	63 ± 1
2.5CBS/3ZnO/3Act8	2.4 ± 0.1	3.4 ± 0.1	21.2 ± 0.8	725 ± 9	63 ± 1
1.5CBS/3ZnO/4Act8	2.2 ± 0.1	2.9 ± 0.1	19.9 ± 1.4	751 ± 15	62 ± 1
3ZnO/4Act8	1.9 ± 0.1	2.2 ± 0.1	17.0 ± 0.4	895 ± 6	60 ± 1
Vulcanizates with CBS and DPG					
CBS/DPG/5ZnO	2.1 ± 0.1	3.1 ± 0.1	19.6 ± 1.6	750 ± 20	62 ± 1
CBS/DPG/5ZnO/2Act8	2.3 ± 0.1	3.2 ± 0.1	20.5 ± 1.8	716 ± 15	63 ± 1
CBS/DPG/3ZnO/3Act8	2.6 ± 0.1	3.4± 0.1	19.3 ± 1.1	682 ± 16	64 ± 1

**Table 5:** Glass transition temperature (T_g_) and mechanical loss factor (tan δ) of SBR vulcanizates.

Composites	T_g_ (°C)	Tan δ at T_g_ (-)	Tan δ at 25 °C (-)	Tan δ at 60 °C (-)
Vulcanizates with CBS				
2.5CBS/5ZnO	−51 ± 1	0.94 ± 0.08	0.09 ± 0.01	0.07 ± 0.01
2.5CBS/5ZnO/2Act8	−47 ± 1	0.80 ± 0.06	0.08 ± 0.02	0.06 ± 0.02
2.5CBS/3ZnO/3Act8	−47 ± 1	0.82 ± 0.05	0.07 ± 0.01	0.06 ± 0.01
1.5CBS/3ZnO/4Act8	−47 ± 1	0.74 ± 0.08	0.09 ± 0.01	0.07 ± 0.01
3ZnO/4Act8	−50 ± 1	0.88 ± 0.07	0.08 ± 0.02	0.08 ± 0.02
Vulcanizates with CBS and DPG				
CBS/DPG/5ZnO	−49 ± 1	0.83 ± 0.08	0.07 ± 0.01	0.06 ± 0.01
CBS/DPG/5ZnO/2Act8	−48 ± 1	0.76 ± 0.06	0.08 ± 0.02	0.06 ± 0.02
CBS/DPG/3ZnO/3Act8	−47 ± 1	0.81 ± 0.05	0.06 ± 0.01	0.05 ± 0.01

**Table 6:** Thermo-oxidative aging factor (A_f_) of SBR vulcanizates.

Composites	A_f_ (-)
Vulcanizates with CBS	
2.5CBS/5ZnO	0.55 ± 0.08
2.5CBS/5ZnO/2Act8	0.19 ± 0.05
2.5CBS/3ZnO/3Act8	0.20 ± 0.06
1.5CBS/3ZnO/4Act8	0.28 ± 0.08
3ZnO/4Act8	0.77 ± 0.09
Vulcanizates with CBS and DPG	
CBS/DPG/5ZnO	0.49 ± 0.05
CBS/DPG/5ZnO/2Act8	0.56 ± 0.08
CBS/DPG/3ZnO/3Act8	0.54 ± 0.08

**Table 7:** Thermal stability of SBR vulcanizates measured by thermogravimetry (TG) (T_5%_, onset temperature of thermal decomposition; T_DTG_, DTG peak temperature; ∆m, total mass loss during thermal decomposition) (SD: T_5%_ ± 1 °C; T_DTG_ ± 1 °C; ∆m ± 0.7%).

Composites	T_5%_ (°C)	T_DTG_ (°C)	∆m_25–600 °C_ (%)	∆m_600–700 °C_ (%)	Residue at 700 °C (%)
Vulcanizates with CBS					
2.5CBS/5ZnO	355	478	75.3	1.1	23.6
2.5CBS/5ZnO/2Act8	352	478	76.0	1.1	22.9
2.5CBS/3ZnO/3Act8	351	479	76.7	1.0	22.3
1.5CBS/3ZnO/4Act8	350	479	77.4	0.9	21.7
3ZnO/4Act8	358	477	76.4	0.9	22.7
Vulcanizates with CBS and DPG					
CBS/DPG/5ZnO	326	477	77.0	1.0	22.0
CBS/DPG/5ZnO/2Act8	333	477	76.8	1.0	22.2
CBS/DPG/3ZnO/3Act8	331	478	77.9	0.9	21.2

**Table 8:** Thermal stability of pure CBS and DPG measured by TG (SD: T_5%_ ± 1 °C; T_DTG_ ± 1 °C; ∆m ± 0.9%).

Accelerator	T_5%_ (°C)	T_DTG_ (1st Step) (°C)	∆m (1st Step) (%)	T_DTG_ (2nd Step) (°C)	∆m (2nd Step) (%)
CBS	219	240	27.3	329	71.6
DPG	198	226	36.4	427	62.9

## Data Availability

The data presented in this study are available on request from the corresponding author.
